# Timely Care: An Audit of Time to Consultation for Patients Rweferred to Older Peoples Service CMHT North Leeds

**DOI:** 10.1192/bjo.2026.11821

**Published:** 2026-06-30

**Authors:** Charlotte Palmer

**Affiliations:** LYPFT, Leeds, United Kingdom

## Abstract

**Aims:**

To assess how many patients were seen within the 15 working days from referral to first appointment within North Leeds OPS CMHT in line with LYPFT guidance and whether electronic documentation and letter to GP were completed in a timely manner. A re-audit was completed after interventions were implemented to assess the effectiveness of these. The interventions implemented were: including resident doctors in the gatekeeping assessment rota; reminder of need to document reasons for delay in assessment in referrals meeting or subsequent contacts and implementing a record of patients not accepted for follow up after assessment with reminders to be sent to assessing staff of need to complete letter within 2 weeks by administration team.

**Methods:**

A review of all referrals to North Leeds OPS CMHT was completed from August to October 2024 and subsequently from August to October 2025. Each patient’s notes were reviewed to extract the outcome of referral. If accepted for assessment; referral date, first appointment date, date of electronic documentation and date of letter to GP sent were extracted. The time intervals between referral and each outcome were subsequently calculated utilising microsoft excel.

**Results:**

For the 2024 cycle the following results were found: 80 referrals were received with 30 referrals accepted for assessment; 70% were seen within 15 working days; 33% had reason for delay documented; all had electronic documentation; 73% had electronic documentation within 24 hours; 87% had letters to GP sent and 33% had GP letter sent within 2 weeks.

For the 2025 cycle the following results were found: 100 referrals were received with 39 referrals accepted for assessment; 87% were seen within 15 working days; 40% had reason for delay documented; 97% had electronic documentation; 61% had electronic documentation within 24 hours; 79% had letters to GP sent and 41% had a GP letter sent within 2 weeks.

**Conclusion:**

Despite an increase in service demands there was an improvement in the number of patients seen within 15 working days with this now sitting above the trust set standard of 80% demonstrating the impact of inclusion of more MDT members for gatekeeping assessments. There were mixed results with regards to timeliness of documentation, this was likely a result of interventions being implemented at a time of greater service pressure resulting in prioritisation of direct patient care above documentation.

